# Regulating Self-Image on Instagram: Links Between Social Anxiety, Instagram Contingent Self-Worth, and Content Control Behaviors

**DOI:** 10.3389/fpsyg.2021.711447

**Published:** 2021-08-25

**Authors:** Richard B. Lopez, Isabel Polletta

**Affiliations:** Department of Psychology, Bard College, Annandale-on-Hudson, NY, United States

**Keywords:** social anxiety, contingent self-worth, Instagram, social media, control

## Abstract

Social media platforms have provided human beings with unprecedented ways to virtually connect with one another, creating a novel and complex arena for psychological research. Indeed, a growing body of research has uncovered links between social media use and various aspects of health and wellbeing. However, relatively little work has examined factors that characterize how people experience and regulate their online selves on particular platforms. In the present study, we recruited a large sample of active Instagram users (*N* = 247; ages 18–58) to complete a questionnaire battery that included measures of participants' social anxiety, their sense of self-worth tied to Instagram use, and specific content control behaviors on the Instagram platform (e.g., editing captions, disabling comments, etc.). Results indicated that participants with higher levels of social anxiety tended to have greater Instagram contingent self-worth, and this was then associated with some content control behaviors, including editing captions and photos and videos when sharing posts. These findings suggest that those who are more socially anxious interact with Instagram differently, and this may arise from self-worth that is wedded to their experiences on the platform. Overall, this work adds to a growing body of research highlighting the benefits and risks of social media use on psychological health.

In today's hyperconnected world, many of our interactions with other human beings are virtual and mediated by social media platforms. Instagram, the popular photo and video sharing app owned by Facebook, boasts over 1 billion active monthly users, or roughly 13% of the world's population. Throughout the COVID-19 pandemic, 69% of people reported that their Instagram use increased the most compared to other apps and platforms (Gothivarekar, 2020). With so much time spent online before and since the pandemic, it is important to consider the psychological effects of social media use, and Instagram use in particular given its prevalence.

Some research to date has examined links between social media use and markers of mental health and wellbeing, especially among young adults and adolescents. Overall, the findings have been mixed, as some studies have found weak or no associations between time spent on social media and multiple health indicators, including depressive symptoms (Heffer et al., 2019) and various measures of well-being and psychological functioning (Orben and Przybylski, 2019). Other studies have found negative associations between social media use and mental health measures, such that increased social media use was correlated more strongly with depressive symptoms and poor self-esteem, particularly among adolescent girls (Kelly et al., 2018) and young women (Sherlock and Wagstaff, 2019). There are several issues with this literature that may account for these inconsistent findings. First, the populations under study have often consisted primarily of adolescents (vs. a wider age range). Second, the measures to estimate social media use are relatively coarse in that they ask people to report their frequency of use, vs. *how* exactly they engage with social media apps and how their engagement reflects psychological processes that promote (or threaten) various aspects of health and wellbeing.

In addition to addressing the above issues, research in this space should also take into consideration the fact that, given the sharp rise in mental health disorders in the past 10–15 years (Twenge et al., 2019), social media users may be at higher risk for psychological disorders, especially anxiety disorders (Vannucci et al., 2017). Anxiety disorders comprise the most common mental health condition in the United States, impacting more than 40 million adults aged 18 or older. Social Anxiety Disorder (SAD) is the second most commonly diagnosed anxiety disorder, after phobias (Anxiety and Depression Association of America). SAD is defined as a “marked fear or anxiety about one or more social situations in which the individual is exposed to possible scrutiny by others” (American Psychiatric Association, 2013). Those with SAD often experience fear of humiliation or negative judgement in social or performative contexts (Heimberg et al., 1995), whereas less socially anxious individuals without an SAD diagnosis do not experience such fear.

A person's social media use and interactions with others on social media platforms, although occurring virtually, are embedded in socially evaluative contexts. Since fear of evaluation is considered a core feature of social anxiety (Weeks et al., 2010), it is likely that highly socially anxious people may be more motivated to engage in behaviors to make a particular (virtual) impression to minimize the likelihood of negative evaluations. Indeed, this is consistent with and an extension of Schlenker and Leary's (1982) Social Anxiety and Self-Presentation (SASP) Model, which posits that someone is more likely to experience social anxiety if they are highly motivated to make a particular impression on others (a “preferred impression”) but doubt their ability to do so (Schlenker and Leary, 1982).

One's propensity to chronically perceive and fear evaluation from others, as is commonly experienced in SAD and those with pre-clinically high levels of social anxiety (Weeks et al., 2010), may also be linked to core intrapersonal processes related to how they see themselves as well as how they maintain their self-image, which has been demonstrated among adolescents (Di Blasi et al., 2015). Additionally, when in a social situation those with SAD report significantly lower implicit and explicit self-esteem compared to controls (Ritter et al., 2013). Contingent self-worth, a related but separate construct from self-esteem, describes people's perceptions of what they need to do or what they need to be or act like in order to attain value and worth as a person (Crocker and Knight, 2005). Self-worth contingencies can show up in many domains, such as rising the corporate ladder to earn accolades from coworkers, or earning high grades in school to maintain a person's sense of intelligence or capabilities (and the value and self-esteem they derive from this self-perception).

In some cases, these contingencies can be motivating by spurring people to behave in ways that are consistent with their goals and values, but the flip side is that they can act as a psychological liability and vulnerability (Crocker et al., 2006), such that those with stronger self-worth contingencies experience more frequent and unpredictable fluctuations in self-esteem, which can potentiate the risk of mental health conditions, including depressive symptoms (Crocker, 2002; Crocker and Knight, 2005). There is one meta-analysis that indicates a link between self-related processes and social media use, showing that those with greater social media usage tended to have lower self-esteem (Liu and Baumeister, 2016). But aside from a study by Kanat-Maymon et al. (2018) examining contingent self-worth and its links to Facebook addiction (Kanat-Maymon et al., 2018) and another by Stefanone et al. (2011) that investigated some aspects of contingent self-worth on Facebook (Stefanone et al., 2011), there has been relatively little work examining social anxiety as a predictor of contingent self-worth and *specific* behaviors people engage in on social media platforms, especially Instagram.

Taken together, the theorizing and finding mentioned above raise several key questions that motivated the present work: how do levels of social anxiety relate to how people present and regulate their self-image on social media platforms, and how might this arise from a person's sense of contingent self-worth while using these platforms? To address these questions, we conducted a study in which we recruited a large and geographically diverse sample of participants on the Prolific platform to take part in an online survey. The survey contained questionnaires that assessed several specific variables of interest, including social anxiety, Instagram contingent self-worth, and specific Instagram content control behaviors, which we operationalize here as ways in which an Instagram user exerts greater control over the content they share on the platform (e.g., by editing and annotating posts; see additional details in Method below).

In light of the questions above and the measures we selected to focus on in this study, we had the following a priori hypotheses^1^: (1) that participants' social anxiety would be associated with Instagram control behaviors, such that more socially anxious individuals would interact with the Instagram platform differently by more readily curating their content and enabling other restrictions, (2) that these results would potentially be accounted for by Instagram contingent self-worth, as those with higher social anxiety would derive more value and self-affirmation from their Instagram use than their less socially anxious counterparts.

## Method

All data and R scripts used for the analyses reported here are available online at the following link: https://osf.io/f8qcs/?view_only=4e002c3b95674f1cb499287a0a4c4728.

### Participants and Power Calculation

We recruited participants using the Prolific platform in order to have a large and geographically diverse sample. There were two inclusion criteria that participants had to meet in order to be eligible to participate in the study: (1) Be between the ages of 18 and 65; and (2) Must have at least one active Instagram account, which was determined by participants responding with “yes” to the question “Do you use Instagram on a regular basis (at least once a month)?” The final sample consisted of 247 adults (118 Females; *M*_age_ = 27.4; *SD*_age_ = 7.95; Age range = 18–58) who reported residing in countries across multiple continents, including Africa (*N* = 2), Asia (*N* = 1), Australia (*N* = 5), Europe (*N* = 216), North America (*N* = 19), and South America (*N* = 1). As far as the racial/ethnic makeup of the sample, a large majority of participants identified as White (211; 85.4%), followed by Asian (11; 4.4%), Black (10; 4%), Mixed Race (10; 4%), and Other/Unspecified (4; 1.6%). All participants provided informed consent in accordance with guidelines set by the Institutional Review Board at Bard College.

We conducted an a priori power analysis using the *pwr* package in R (Champely, 2020) by specifying a small effect size (*r* = 0.2), 90% power, and alpha = 0.05 for a two-sided test. The resulting recommended sample size was 258. Our final sample size of 247 participants indicated that our analyses would be sufficiently powered to detect at least small or modest effect sizes, in terms of associations between variables of interest.

### Measures

#### Social Anxiety

Social anxiety was assessed via the Social Anxiety Questionnaire for Adults (SAQ-A), a validated measure of social anxiety (Caballo et al., 2012). The SAQ-A consists of 30 items preceded by a prompt asking respondents to rate their level of “unease, stress, or nervousness” in various social situations using a likert scale ranging from 1 (“Not at all or very slight”) to 5 (“Very high or extremely high”). Items follow a five-factor structure: (1) speaking in public/talking with people in authority (e.g., “Talking to a superior or a person in authority”), (2) interactions with the opposite sex (e.g., “Being asked out by a person I am attracted to”), (3) assertive expression of annoyance, disgust or displeasure (e.g., “Having to ask a neighbor to stop making noise”), (4) criticism and embarrassment (e.g., “Being criticized”), and (5) interactions with strangers (e.g., “Attending a social event where I know only one person”) (Caballo et al., 2012). Scale items showed high internal consistency, Cronbach's alpha = 0.95. Responses to all items were summed so that each participant had one score reflecting their overall tendency to experience social anxiety.

#### Instagram Contingent Self-Worth

Because no Instagram Contingent Self-Worth scale existed when we designed and prepared for this study, we adapted items and wording from Crocker, Luhtanen, Cooper and Bouvrette (2003) Contingencies of Self-Worth Scale, specifically the subscale representing contingent self-worth based on others' approval (Crocker et al., 2003). We administered a brief, four-item survey in which we asked participants to indicate level of agreement with the following statements, using a 5-point likert scale (1 = Strongly disagree; 2 = Disagree; 3 = Neutral; 4 = Agree; 5 = Strongly agree): (1) “When I get a lot of likes and new followers on my Instagram, my self-esteem increases;” (2) “I feel worthwhile when others like or comment on my Instagram posts;” (3) “When my Instagram posts or comments go unnoticed, I feel badly about myself;” and (4) “My self-esteem depends on how popular and active my Instagram profile is.” Although we did not intend this survey to have robust psychometric properties of longer scales, the Cronbach's alpha of 0.80 indicated sufficient internal consistency, so we summed the responses from the four items to create one Instagram contingent self-worth score for each participant.

#### Instagram Control Behaviors

Instagram offers users a variety of tools for users to carefully curate the content they create and share on the platform. Editing tools for photographs and videos include image rotation and cropping, filters and effects, and the ability to compare edited images to original images before posting. Users can spend as much time as they want editing their content before posting and they also have the opportunity to edit a post's captions after the fact. Comments are a popular addition to a post and can be written by the individual posting the content or by followers. Both public and private accounts allow for comments. However, users can filter, delete, or disable comments on a post-by-post basis. Users can also turn off commenting to prevent others from commenting on an individual post. Given these features, we administered a brief 3-item survey in which we specifically asked about the following: (1) “Regarding content that you post on Instagram, how frequently do you disable comments for individual posts?”; (2) “Of the content that you post on Instagram, approximately how long do you edit and annotate photos/videos you share in posts or stories?”; and (3) “How often do you edit the captions of your posts after you have posted?” Participants responded to each of these items using a 1–5 likert scale. Originally, we summed responses across the three items in order to have one score per participant reflecting propensity to engage in Instagram content control behaviors. However, internal reliability was low (Cronbach's alpha = 0.453), so for all subsequent analyses we decided examine the three behaviors separately (vs. as an aggregate measure).

### Analytic Procedure

First, we computed all pairwise correlations between the three categories of the variables of interest [i.e., social anxiety (reflected by SAQ-A scores), Instagram contingent self-worth, and several Instagram content control behaviors]. Next, we used the *lavaan* package in R (Rosseel, 2012) to specify three mediation models predicting the aforementioned Instagram content control behaviors, respectively. In each model, social anxiety served as the predictor variable and Instagram contingent self-worth was the intervening variable. Participants' age, sex, and daily Instagram screen time were included as covariates in all models. Daily Instagram screen time served as a proxy to capture participants' overall Instagram use/potential overuse, which has previously been associated with negative life impacts (e.g., Zheng and Lee, 2016). Bootstrapping with 5,000 iterations was performed to estimate uncertainty (confidence intervals and standard errors) around all model estimates.

Although these mediations models were fit using cross-sectional data, the variables can be conceptualized along a distal-to-proximal continuum in the order in which they appear in the mediation [i.e., given the above cited work, social anxiety serving as the broadest and most distal predictor variable (X), followed by Instagram contingent self-worth as less distal/more proximal (M), followed by specific Instagram content control behaviors, such as editing captions after one has posted on IG (Y)]. Although not ideal, this is an acceptable approach one can take to assess plausible mediation processes with cross-sectional data (Shrout and Bolger, 2002). Second, it is reasonable to assume that an individual's level of social anxiety temporally proceeds their exposure to social media, at least in some instances, given that the onset of social anxiety disorder can be as early as 13–14 (Anxiety and Depression Association of America). To confirm this, we computed model fit indices (AIC) and found that having social anxiety as the predictor variable and Instagram contingent self-worth as the mediating variable for all models led to better model fit, as indicated by markedly lower AIC values (see below).

## Results

### Pairwise Correlations

Some variables of interest were significantly correlated with one another, with the directionality of the associations in line with our predictions and hypotheses. For example, there was a significant positive association between social anxiety and Instagram contingent self-worth, *r* = 0.19 (95% CI: 0.07, 0.31), *t*(245) = 3.02, *p* = 0.003. Additionally, there was a significant positive relationship between Instagram contingent self-worth and all three Instagram content control behaviors: for disabling comments, *r* = 0.14 (95% CI: 0.01, 0.26), *t*(245) = 2.28, *p* = 0.024; for time spent editing captions, *r* = 0.25 (95% CI: 0.13, 0.36), *t*(245) = 4.01, *p* < 0.001; and for time spent editing photos and videos in posts, *r* = 0.26 (95% CI: 0.14, 0.37), *t*(245) = 4.14, *p* < 0.001. As far as links between social anxiety and control behaviors, there was a significant association between social anxiety scores and time spent editing photos and videos, *r* = 0.14 (95% CI: 0.01, 0.26), *t*(245) = 2.14, *p* = 0.03, but correlations with the other two Instagram control behaviors (disabling comments and time spent editing captions) were not significant, *p*'s ≥ 0.38. See Table 1 for all pairwise correlations and their confidence intervals.

**Table 1:** Pairwise correlations between all measures of interest.

**Variable**	**1**	**2**	**3**	**4**
1. Social anxiety (SAQ score)				
2. Instagram contingent self-worth	0.19^**^			
	[0.07, 0.31]			
3. Disabling comments on posts	0.00	0.14^*^		
	[−0.12, 0.13]	[0.02, 0.26]		
4. Editing/annotating photos and videos in shared posts and stories	0.06	0.25^**^	0.19^**^	
	[−0.07, 0.18]	[0.13, 0.36]	[0.07, 0.31]	
5. Editing captions of posts	0.14^*^	0.26^**^	0.12	0.34^**^
	[0.01, 0.26]	[0.14, 0.37]	[−0.01, 0.24]	[0.22, 0.44]

*Values in square brackets indicate the 95% confidence interval for each correlation*.

* *Indicates p < 0.05*.

** *Indicates p < 0.01*.

### Results From Mediation Models Testing Indirect Paths Between Social Anxiety and Instagram Control Behaviors

**Figure 1:**
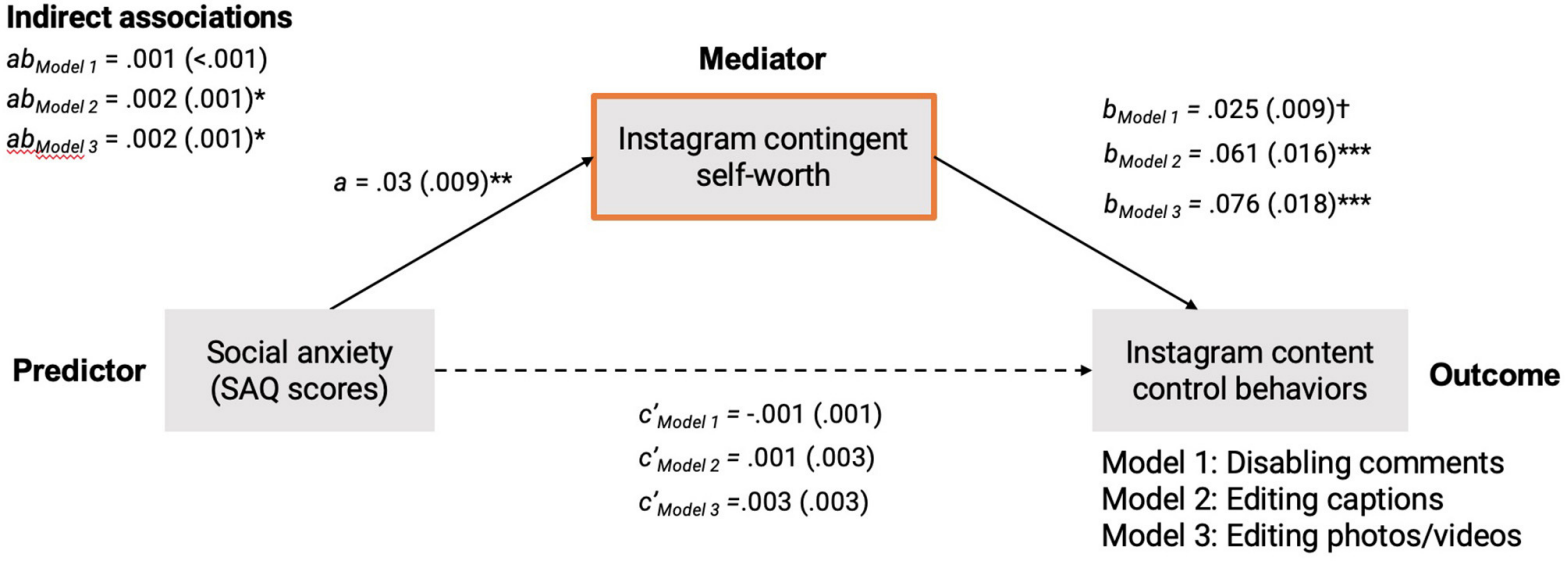
Path diagram depicting all direct and indirect paths represented by the three mediation models predicting Instagram content control behaviors as a function of social anxiety levels and Instagram contingent self-worth. Estimates of indirect associations are designated by *ab*. Dashed lines indicate direct paths between the predictor and the outcome measures. Potential influences of age, sex, and daily Instagram screen time (not shown) are controlled for in all paths. All numbers indicate unstandardized path coefficients, with estimates of standard error in parentheses. ^†^Indicates *p* < 0.10; ^*^indicates *p* < 0.05; ^**^indicates *p* < 0.01; ^***^indicates *p* < 0.001. *P*-values for indirect associations are FDR-adjusted.

With social anxiety as the predictor variable and Instagram contingent self-worth as the mediating variable, the three mediation models specified above accounted for 2.6–5.7%^2^ of the variance in the extent to which participants engaged in Instagram control behaviors (i.e., disabling comments, editing captions, and editing photos/videos). Although there were no significant direct effects, there were two significant indirect associations between social anxiety and Instagram control behaviors via greater Instagram contingent self-worth. This was true in the model predicting caption editing, *b* = 0.002 (95% bootstrapped CI: 0.001, 0.003), *z* = 2.42, *p*_adjusted_ = 0.048,^3^ 66.7% mediation, and photo/video editing when posting, *b* = −0.03 (95% bootstrapped CI: −0.05, −0.01), *z* = −2.70, *p*_adjusted_ = 0.033, 33.3% mediation. There was no significant indirect association between social anxiety and comment disabling, *b* = 0.001 (95% bootstrapped CI: 0.000, 0.002), *z* = 1.56, *p*_adjusted_ = 0.357. See Figure 1 depicting path coefficients from all three mediation models.

Lastly, the a priori model specifications exhibited better model fit, as indicated by lower AIC values, than an alternative model specification with the predictor and mediator switched (i.e., Instagram contingent self-worth as the predictor and social anxiety as the mediator), AIC_model1−original_ = 1708.887, AIC_model1−alternate_ = 2617.468; AIC_model2−original_ = 1859.631, AIC_model2−alternate_ = 2761.207; and AIC_model3−original_ = 1945.560, AIC_model3−alternate_ = 2850.411.

## Discussion

The present findings are generally consistent with our hypotheses, namely: that people's levels of social anxiety are in fact related to self-worth tied to specific aspects of their Instagram use, and that this has implications for how socially anxious individuals engage with the platform. Specifically, indirect paths in mediation models revealed that those who reported being more anxious in social situations tended to have higher Instagram contingent self-worth, which was correlated with multiple Instagram content control behaviors, namely spending more time editing captions and annotating photos and videos. All of these results were observed in models that adjusted for participants' age, sex, and Instagram daily screen time, suggesting that above and beyond the potential influence of these variables, social anxiety is an important factor when characterizing Instagram users' sense of self-worth (tied to the platform) and specific content control behaviors.

The fact that these relationships held while controlling for Instagram screen time suggests that it is not that socially anxious individuals merely spend more overall time on Instagram (in this sample, there was no correlation, *r* = 0.04), which is consistent with recent work showing weak associations (at best) between screen time measures and indicators of mental health and wellbeing (e.g., Orben and Przybylski, 2019). Rather, those who are socially anxious reported greater worth and higher self-esteem when experiencing recognition and affirmation from others on the platform (e.g., receiving likes and gaining new followers), and this then led them to interact with the platform differently by curating the content they post to a greater degree than less socially anxious individuals. Moreover, given that the social anxiety measure used here (SAQ-A by Caballo et al., 2012) reflects unease with regard to *in-person* interactions, we speculate that the psychological processes that underlie this interpersonal anxiety may also be involved in virtual interactions people have on Instagram.

There are strengths and weaknesses of the present work worth discussing. First, with regard to strengths, the findings shed light on important individual difference factors at play when people engage with social media platforms. Rather than coarse screen time measures within or across platforms, we focused on one social media platform (Instagram) and outcome measures reflecting content control behaviors that are specific to Instagram.^4^ Moreover, we used a novel measure of contingent self-worth as it relates to a person's Instagram activity. This adds to a growing body of literature that has recently begun to highlight the benefits and risks of social media use as a function of specific intrapersonal processes. For example, one study examined domain general self-worth and its links to Facebook addiction and screen time (Kanat-Maymon et al., 2018). Lastly, the participant sample here is relatively large and geographically—and to some degree socio-culturally—diverse, which increases confidence that the findings are generalizable to a broader population.

Regarding limitations, the first and perhaps most important limitation to mention is that we employed mediation models using cross-sectional data. Although we had directional hypotheses about the variables of interest, the models showed better fit (compared to models with X and M switched), and the variables followed a plausible distal-to-proximal continuum (see above mentioned rationale in Method), the study was nonetheless correlational and measurements were collected only at one time point. Thus, any strong inferences about causality cannot be made with the present data. For example, instead of social anxiety having downstream, causal impacts on self-worth and content control behaviors, it could be that one's experiences while using Instagram over time could feed back and alter social anxiety levels. Experimental or longitudinal designs will be better suited to tease out the causal relations between these variables. We also recommend that future research considers testing the role of other potential mediating or moderating variables that could also play a role in predicting self-worth and Instagram use patterns, including narcissism (Campbell et al., 2002; Paramboukis et al., 2016) and upward social comparison (Chua and Chang, 2016).

Another caveat is that all findings were derived from self-report measures, so there is the potential of various reporting biases at play that could have affected the results (e.g., participants may under- or over-estimate the time they spend engaged in content control behaviors on Instagram). To mitigate this issue, follow up work would benefit from having additional and more objective app-level or phone-level data and determining whether such data are consistent with self-reported measures. Furthermore, due to lack of an extant and valid measure for Instagram contingent self-worth, we adapted an existing self-worth subscale by Crocker et al. (2003). We encourage other researchers to use and validate this particular measure in future work, assess convergent/discriminant validity with related constructs such as feedback sensitivity people experience on other social media platforms (e.g., Facebook; Hart et al., 2015), and also consider developing additional measures that capture control behaviors on Instagram, especially given the constantly changing functionality and features of the app. Lastly, while our sample was diverse along some demographic dimensions (e.g., not limited to adolescents or college-aged convenience samples, some geographical diversity), most participants in the sample identified as White, so follow-on studies will need to test whether the present findings generalize to a more racially and ethnically diverse population.

To conclude, the current study examined different aspects of people's experiences with Instagram, with a focus on social anxiety and how that might relate to varying engagement with the platform arising from specific psychological constructs, namely Instagram contingent self-worth. Given the results described above, it appears that for people who struggle with social anxiety, part of their self-worth is tied to positive recognition from other Instagram users, which then correlates more frequent content control behaviors. Indeed, researchers and clinicians might want to consider taking people's social anxiety, contingent self-worth, and app-level behaviors when determining whether social media use is problematic for one's psychological health and wellbeing (or not). Such an approach would reflect a more nuanced understanding of the numerous and complex ways in which we perceive and conduct ourselves online.

## Data Availability Statement

The datasets presented in this study can be found in online repositories. The names of the repository/repositories and accession number(s) can be found in the article/supplementary material.

## Ethics Statement

The studies involving human participants were reviewed and approved by Institutional Review Board, Bard College. The patients/participants provided their written informed consent to participate in this study.

## Author Contributions

RL and IP conceived the study design and hypotheses. RL administered the study and conducted data analysis. Both authors worked on the manuscript.

## Conflict of Interest

The authors declare that the research was conducted in the absence of any commercial or financial relationships that could be construed as a potential conflict of interest.

## Publisher's Note

All claims expressed in this article are solely those of the authors and do not necessarily represent those of their affiliated organizations, or those of the publisher, the editors and the reviewers. Any product that may be evaluated in this article, or claim that may be made by its manufacturer, is not guaranteed or endorsed by the publisher.

## Footnotes

^1^Findings observed in a smaller pilot study using an independent sample of college students (unpublished data) informed the research questions and hypotheses tested in the present study.

^2^Adjusted *R*-square value.

^3^To control for potentially inflated Type I error rate, we applied the Benjamini–Hochberg procedure to the three p-values corresponding to the three indirect effects of interest; this approach controls the false discovery rate while maintaining statistical power (Benjamini and Hochberg, 1995).

^4^Although we controlled for time spent on Instagram in all models, as an additional check we separately computed pairwise correlations between the screen time measure (average daily Instagram screen time, in hours) and variables of interest, and there were no significant correlations, −0.11 ≤ *r*'s ≤ 0.09, *p*'s ≥ 0.10.
